# Plasma fatty acids and the risk of metabolic syndrome in ethnic Chinese adults in Taiwan

**DOI:** 10.1186/1476-511X-10-33

**Published:** 2011-02-21

**Authors:** Kuo-Liong Chien, Chia-Lun Chao, Chen-Hong Kuo, Hung-Ju Lin, Pi-Hua Liu, Pei-Rony Chen, Hsiu-Ching Hsu, Bai-Chin Lee, Yuan-Teh Lee, Ming-Fong Chen

**Affiliations:** 1 Institute of Epidemiology & Preventive Medicine, College of Public School, National Taiwan University, Taipei, Taiwan; 2 Department of Internal Medicine, National Taiwan University Hospital, Taipei, Taiwan; 3 School of Medicine, Chang-Gun University, Lin-Kou, Taoyuan, Taiwan; 4 Department of Nutrition, National Taiwan University Hospital, Taipei, Taiwan; 5 Chinese Medical University Hospital, Taichung, Taiwan

## Abstract

**Background:**

Evidence of predictive power of various fatty acids on the risk of metabolic syndrome was scanty. We evaluated the role of various fatty acids, including saturated fat, monounsaturated fat, transfat, n-6 fatty acid, eicosapentaenoic acid (EPA) and docosahexaenoic acid (DHA), for the risk of the metabolic syndrome in Taiwan.

**Results:**

A nested case-control study based on 1000 cases of metabolic syndrome and 1:1 matched control subjects. For saturated fat, monounsaturated fat and transfat, the higher the concentration the higher the risk for metabolic syndrome: participants in the highest quintile had a 2.22-fold (95% confidence interval [CI], 1.66 to 2.97) higher risk of metabolic syndrome. In addition, the participants in higher EPA quintiles were less likely to have the risk of metabolic syndrome (adjusted risk, 0.46 [0.34 to 0.61] for the fifth quintile). Participants in the highest risk group (low EPA and high transfat) had a 2.36-fold higher risk of metabolic syndrome (95% CI, 1.38 to 4.03), compared with those in the lowest risk group (high EPA and low transfat). For prediction power, the area under ROC curves increased from 0.926 in the baseline model to 0.928 after adding fatty acids. The net reclassification improvement for metabolic syndrome risk was substantial for saturated fat (2.1%, *P *= 0.05).

**Conclusions:**

Plasma fatty acid components improved the prediction of the metabolic syndrome risk in Taiwan.

## Introduction

Identifying dietary factors for the development of type 2 diabetes and metabolic syndrome is essential for primary prevention [1,2]. Dietary intake habits of fatty acids, including consumption of foods with high saturated fat and high transfat contents, are associated with insulin resistance and hyperlipidemia [3]. In addition, transfat has frequently been reported to be risk factors for cardiovascular diseases [4,5], and the evidence for monounsaturated fats was inconclusive[6]. In contrast, marine-derived fatty acids, such as eicosapentaenoic acid (EPA) and docosahexaenoic acid (DHA) are inversely related to type 2 diabetes due to reduced inflammation and increased insulin sensitivity [7]. However, although there have been many studies about the association of specific fatty acids and the risk of cardiovascular diseases, there have only been a limited number of integrated comparison studies. Previous studies on fatty acids have focused on specific fatty acids only, without considering all of the fatty acids together [8-10]. In addition, the performance measures of each fatty acid have not been evaluated extensively, and clinical situations not considered simultaneously. Therefore, the aim of this study was to evaluate the specific role of various fatty acids, including saturated fat, monounsaturated fat, transfat, n-6 fatty acid, EPA and DHA, for the risk of the metabolic syndrome among ethnic Chinese adults in Taiwan. In addition, we evaluated the performance measures of these fatty acids and investigated the joint effect of two fatty acids together to test the prediction for the risk of metabolic syndrome.

## Results

Table 1 presents the clinical, biochemical distribution, and fatty acid profiles of the study participants, specified by metabolic syndrome status. Compared with the control subjects, the case participants with metabolic syndrome were more likely to have a higher blood pressure, body mass index, waist circumference, total and LDL cholesterol, triglycerides, fasting glucose, uric acid, glomerular filtration rate, and a lower HDL cholesterol level. The case patients were more likely to smoke and less likely to exercise regularly. Due to the matched factors, age and gender were similar between the two groups. With regards to the absolute amount of fatty acids, participants with metabolic syndrome were more likely to have higher levels of saturated fatty acid, n-6 fatty acid, polyunsaturated fatty acid, and transfat. However, after adjusting for total fatty acid intake, the case participants with metabolic syndrome tended to have higher levels of saturated fat, monounsaturated fatty acid, and lower n-6 fatty acid, n-3 fatty acid, marine fatty acid, polyunsaturated fatty acid, and EPA and DHA concentrations compared with the control subjects. The age and gender adjusted Spearman correlation coefficients between the biochemical and fatty acid concentrations are shown in Additional file 1 Table S1. The correlations for absolute amount and relative percentage of fatty acids were different: for the absolute amount of fatty acids, most coefficients were positive for triglycerides, ranging from 0.11 for marine fatty acid to 0.74. However, the coefficients changed modestly for total fat percentage: marine fatty acid, EPA and DHA were inversely related to waist circumference (ranging from -0.10 to -0.16) and triglycerides (-0.24 to -0.29), and positively related to HDL cholesterol (0.16 to 0.21). Other clinical variables, including blood pressure, total cholesterol, LDL cholesterol, uric acid, fasting glucose, and glomerular filtration rate, were not significantly associated with EPA and DHA.

**Table 1:** Basic characteristics and fatty acid distributions of the study participants, specified by metabolic syndrome status

	No metabolic syndrome n = 986		Metabolic syndrome, n = 1000		
	**Freq**	**%**	**Freq**	**%**	
Gender					0.67
Women	348	35.3	362	36.2	
Men	638	64.7	638	63.8	
Smoking	139	14.1	185	18.5	0.008
Drinking	575	58.3	571	57.1	0.58
Exercise	549	55.7	502	50.2	0.014

	Mean	Std Dev	Mean	Std Dev	*P*
Age, yr	54.4	10.8	54.9	10.7	0.32
Systolic blood pressure, mmHg	122.7	14.8	133.2	14.8	< .0001
Diastolic blood pressure, mmHg	72.9	9.8	79.2	10.2	< .0001
Body mass index, kg/m^2^	22.8	2.3	27.1	3.0	< .0001
Waist, cm	79.2	6.4	94.3	7.0	< .0001
Total cholesterol, mg/dL	202.7	36.4	206.8	36.8	0.012
Triglycerides, mg/dL	115.4	74.0	178.3	104.5	< .0001
HDL cholesterol, mg/dL	43.1	9.7	38.5	7.0	< .0001
LDL cholesterol, mg/dL	116.3	31.5	124.9	32.7	< .0001
Uric acid, mg/dL	6.2	1.5	6.6	1.6	< .0001
Fasting glucose, mg/dL	93.1	22.2	105.0	28.1	< .0001

Fatty acid, mg/dL					
Saturated fat	1713.0	512.9	1982.9	682.8	< .0001
MUFA	616.3	246.5	728.9	285.9	< .0001
Trans fatty acid	267.4	106.7	322.3	158.9	< .0001
PUFA	1488.2	347.9	1542.7	361.7	0.0006
N-6 fatty acids	1297.4	313.4	1350.0	325.1	0.0003
N-3 fatty acids	190.8	60.8	192.7	58.9	0.48
Marine fatty acids	132.7	52.4	131.6	50.3	0.64
EPA	21.5	14.5	20.3	12.5	0.06
DHA	111.2	42.7	111.3	41.9	0.97
Total fat amount	40.8	10.7	45.8	13.2	< .0001
Fatty acid, % total fatty acids					
Saturated fat	41.8	4.4	43.0	4.6	< .0001
MUFA	14.8	2.6	15.7	2.6	< .0001
Trans fatty acid	6.52	1.84	6.95	2.00	< .0001
PUFA	36.8	4.7	34.4	4.8	< .0001
N-6 fatty acids	32.1	4.2	30.0	4.2	< .0001
N-3 fatty acids	4.76	1.37	4.34	1.21	< .0001
Marine fatty acids	3.32	1.23	2.97	1.09	< .0001
EPA	0.54	0.37	0.46	0.28	< .0001
DHA	2.78	0.99	2.51	0.91	< .0001

Abbreviations: MUFA: monounsaturated fatty acid; PUFA: polyunsaturated fatty acid; EPA: eicosapentaenoic acid; DHA: docosahexaenoic acid

According to the quintiles of various fatty acids, including saturated fat, monounsaturated fat, transfat, n-6 fatty acid, and EPA and DHA concentrations in the control subjects, we examined the adjusted odds ratios and 95% confidence intervals (CI), and tested for trend for the risk of metabolic syndrome (Table 2). For saturated fat, monounsaturated fat and transfat, the higher the concentration, the higher the risk for the metabolic syndrome. Compared with those in lower saturated fat quintiles, participants in the highest quintile had a 2.22-fold increased risk (95% confidence interval [CI], 1.66 to 2.97, *P *< 0.0001) of having the metabolic syndrome. The effects persisted after multiple adjustments. Similar positive associations existed for monounsaturated fat and transfat: the adjusted risks in the participants in the fifth quintile were 1.89 (95% CI, 1.18 to 3.01) for monounsaturated fat and 1.53 (95% CI, 0.99 to 2.35, test for trend, *P = *0.002) for transfat. However, an inverse association was found for n-6 fatty acid, EPA and DHA. Compared with those in the lowest EPA quintile, the participants in higher EPA quintiles were less likely to have a risk of metabolic syndrome (age and gender adjusted risk, 0.62 [95% CI, 0.47 to 0.81] for the second, 0.60 [0.46 to 0.79] for the third, 0.60 [0.45 to 0.78] for the fourth, and 0.46 [0.34 to 0.61] for the fifth quintile, test for trend, *P *< 0.0001). The protective effects of EPA were still persistent after multiple adjustments. Similar patterns were found for DHA levels. Compared with the first DHA quintile, the multivariate adjusted risks for consequent quintiles were 0.87 (0.58 to 1.29), 0.65 (0.43 to 1.00), 0.51 (0.33 to 0.78) and 0.54 (0.35 to 0.84), respectively. The estimated risk for continuous variables, specified by concentration as a unit or by one standard deviation unit, showed various standardized coefficients, the absolute values ranging from 0.15 for saturated fat to 0.29 for n-6 fatty acid (Additional file 1 Table S2).

**Table 2:** The numbers of the case control subjects according to fatty acid quintiles and adjusted odds ratios in the study participants

	Q1	Q2			Q3			Q4			Q5			*P*, Trend
Saturated fat, % total fatty acids														
Range	< 38.0	38.0-40.1			40.1-42.5			42.5-45.7			> = 45.7			
Median	36.6	39.2			41.2			44.1			48.3			
Control	195	196			196			197			195			< .0001
Cases	127	168			221			201			281			
Odds ratio		OR	95% CI		OR	95% CI		OR	95% CI		OR	95% CI		
Model 1	1	1.31	0.97	1.78	1.75	1.30	2.34	1.57	1.16	2.11	2.22	1.66	2.97	< .0001
Model 2	1	1.05	0.67	1.64	1.48	0.97	2.28	1.20	0.78	1.84	1.94	1.27	2.96	0.002
Model 3	1	1.03	0.65	1.63	1.33	0.85	2.06	1.03	0.66	1.60	1.68	1.08	2.62	0.025

MUFA, % total fatty acids														
Range	< 12.6	12.6-14.1			14.1-15.4			15.4-16.9			> = 16.9			
Median	11.5	13.5			14.7			16.2			18.2			
Control	195	196			197			196			195			< .0001
Cases	117	150			161			256			314			
Model 1	1	1.27	0.93	1.74	1.38	1.01	1.89	2.22	1.65	2.99	2.76	2.06	3.70	< .0001
Model 2	1	1.26	0.80	1.99	1.01	0.64	1.59	1.89	1.22	2.91	2.12	1.37	3.26	< .0001
Model 3	1	1.14	0.71	1.83	0.94	0.59	1.51	1.78	1.14	2.80	1.89	1.18	3.01	0.001

Transfat, % total fatty acids														
Range	< 5.2	5.2-5.9			5.9-6.7			6.7-7.7			> = 7.7			
Median	4.6	5.5			6.3			7.2			8.7			
Control	195	197			194			197			196			< .0001
Cases	147	147			178			222			303			
Model 1	1	0.99	0.73	1.34	1.23	0.91	1.65	1.50	1.12	2.00	2.05	1.55	2.72	< .0001
Model 2	1	0.88	0.56	1.37	1.18	0.77	1.83	1.81	1.20	2.75	1.68	1.11	2.53	0.0003
Model 3	1	0.81	0.51	1.29	1.09	0.69	1.72	1.65	1.07	2.54	1.53	0.99	2.35	0.002

N-6 fatty acids, % total fatty acids														
Range	< 28.7	28.7-31.1			31.1-33.3			33.3-35.8			> = 35.8			
Median	26.4	29.9			32.2			34.4			37.4			
Control	195	196			196			196			196			< .0001
Cases	363	225			181			159			70			
Model 1	1	0.62	0.48	0.80	0.50	0.38	0.65	0.43	0.33	0.57	0.19	0.14	0.27	< .0001
Model 2	1	0.83	0.57	1.21	0.65	0.44	0.96	0.55	0.38	0.82	0.31	0.20	0.49	< .0001
Model 3	1	0.84	0.57	1.24	0.69	0.46	1.04	0.64	0.42	0.97	0.36	0.22	0.59	< .0001

EPA, % total fatty acids														< .0001
Range	< 0.28	0.28-0.38			0.38-0.52			0.52-0.76			> = 0.76			
Median	0.22	0.33			0.44			0.62			0.94			
Control	196	196			196			196			195			
Cases	299	186			185			186			142			
Model 1	1	0.62	0.47	0.81	0.60	0.46	0.79	0.60	0.45	0.78	0.46	0.34	0.61	< .0001
Model 2	1	0.61	0.41	0.92	0.51	0.34	0.76	0.67	0.45	0.99	0.51	0.33	0.77	0.013
Model 3	1	0.60	0.39	0.91	0.51	0.34	0.78	0.68	0.45	1.03	0.51	0.33	0.80	0.028

DHA, % total fatty acids														
Range	< 1.97	1.97-2.45			2.45-2.89			2.89-3.56			> = 3.56			
Median	1.6	2.2			2.7			3.2			4.0			
Control	96	196			195			197			195			
Cases	281	256			167			161			133			
Model 1	1	0.89	0.69	1.16	0.58	0.44	0.77	0.55	0.42	0.73	0.45	0.34	0.60	< .0001
Model 2	1	0.97	0.66	1.43	0.68	0.45	1.02	0.54	0.36	0.82	0.58	0.38	0.89	0.001
Model 3	1	0.90	0.60	1.34	0.69	0.45	1.05	0.55	0.35	0.85	0.59	0.38	0.92	0.003

Model 1: adjusted for age and gender

Model 2: additional body mass index, smoking, drinking and exercise

Model 3: additional LDL cholesterol, systolic and diastolic blood pressure, uric acid, fasting glucose levels and total fat amount in plasma

Abbreviations see Table 1

To test the performance measures of different fatty acid profiles, we estimated the area under the receiver operative characteristics (ROC) curves (AUCs) in the baseline model, and tested the incremental values after adding one fatty acid (Table 3). If treated as quintile values, the AUC increased from 0.926 in the baseline model to 0.928 in the additional n-6 fatty acid model (*P *for difference among 7 models, 0.009). The reclassification tables with and without EPA and transfat are shown in Additional file 1 Table S3, and the performance measure values are listed in Table 4. The net reclassification improvement was substantial for saturated fat (2.1%, 95% CI, 0.0% to 4.2%, *P *= 0.05), and the integrated discriminative improvement values for fatty acids were significant, ranging from 0.2% for saturated fat and DHA to 0.7% for monounsaturated fat and n-6 fatty acid (all *P *< 0.05).

**Table 3:** Area under the ROC curves comparison with the base model

	AUC	95% CI		AUC	95% CI	
Base model	0.926	0.915	0.936			

Additional for:						
	Continuous variables *			Quintile variables **		
Saturated fat, % fat	0.926	0.915	0.937	0.927	0.916	0.937
MUFA, % fat	0.927	0.916	0.937	0.928	0.917	0.939
Transfat, % fat	0.926	0.915	0.937	0.927	0.917	0.938
N-6 fatty acids, % fat	0.927	0.916	0.938	0.928	0.917	0.938
EPA, % fat	0.926	0.916	0.937	0.927	0.916	0.938
DHA, % fat	0.926	0.916	0.937	0.926	0.915	0.937

* test for 7 models, p = 0.08

** test for 7 models, p = 0.009

The base model included: age, gender, body mass index, smoking, drinking and exercise, LDL cholesterol, systolic and diastolic blood pressure, uric acid, fasting glucose levels and total fat amount in plasma

AUC: area under the ROC curve; CI: confidence interval

**Table 4:** Performance measures of the models including various fatty acid levels

	NRI, %	95% CI		P	IDI, %	95% CI		P
Quintile variables								
Saturated fat, % fat	2.1	0.0	4.2	0.05	0.2	0.0	0.5	0.036
MUFA, % fat	2.2	-0.4	4.8	0.09	0.7	0.3	1.0	0.0001
Transfat, % fat	1.7	-0.8	4.2	0.17	0.5	0.2	0.9	0.001
N-6 fatty acids, % fat	1.5	-0.9	3.9	0.22	0.7	0.3	1.0	0.0001
EPA, % fat	1.4	-0.9	3.7	0.24	0.4	0.1	0.7	0.002
DHA, % fat	0.2	-1.5	1.9	0.81	0.2	0.0	0.4	0.046

Continuous variables								
Saturated fat, % fat	0.7	-1.0	2.4	0.42	0.1	-0.1	0.3	0.09
MUFA, % fat	1.4	-0.5	3.3	0.15	0.3	0.1	0.5	0.002

Transfat, % fat	0.3	-1.3	1.9	0.71	0.1	-0.1	0.3	0.08
N-6 fatty acids, % fat	1.6	-0.7	3.9	0.17	0.5	0.2	0.8	0.001
EPA, % fat	1.7	-0.2	3.6	0.08	0.2	0.0	0.5	0.022
DHA, % fat	1.3	-0.6	3.2	0.18	0.2	0.0	0.4	0.031

NRI cutoff points: 0.1, 0.5, and 0.9;

Abbreviations: MUFA: monounsaturated fatty acid; PUFA: polyunsaturated fatty acid; EPA: eicosapentaenoic acid; DHA: docosahexaenoic acid; NRI: net reclassification improvement; IDI: integrated discrimination improvement; CI, confidence interval ;

**Figure 1:**
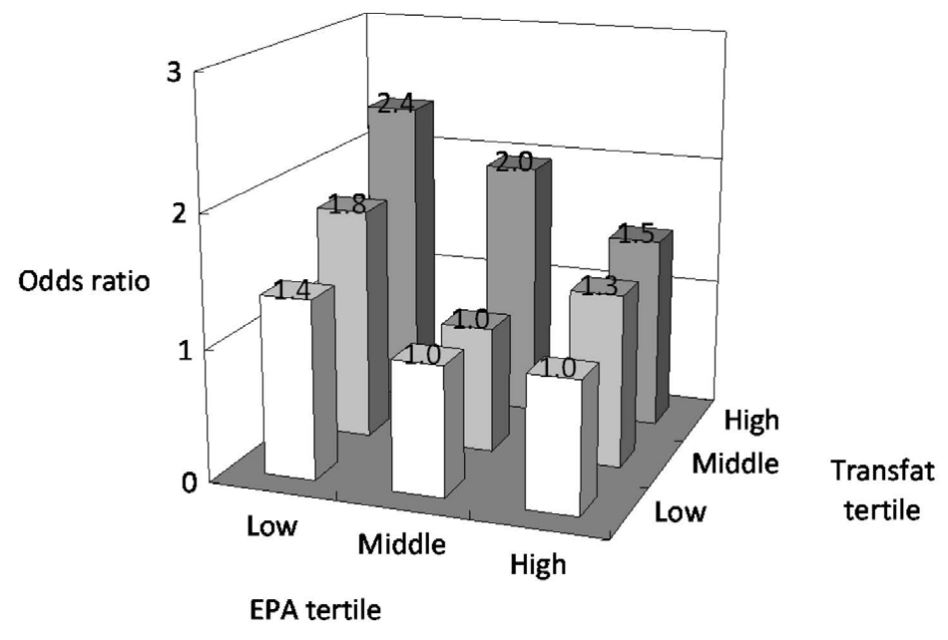
**Odds ratios according to tertiles of EPA and trans fat concentrations, adjusted for age, gender, BMI, smoking, drinking and exercise, LDL cholesterol, systolic and diastolic blood pressure, uric acid, fasting glucose levels and total fat amount in plasma**. Cutoff points for tertiles of EPA were <0.35, 0.35-0.58, and >0.58% fat. Cutoff points for tertiles of transfat were <5.7, 5.7-7.1, and >7.1% fat.

In mutually adjusted models for two fatty acids, most fatty acids remained significant and strong risk factors for metabolic syndrome (Table 5). For example, the adjusted odds ratio of the highest EPA quintile was 0.55 (95% CI, 0.35 to 0.86) and that of the highest saturated fat quintile 1.56 (95% CI, 1.00 to 2.45). In joint analyses, participants in the highest risk group (low EPA and high transfat) had a 2.4-fold higher risk of metabolic syndrome (adjusted odds ratio, 2.36, 95% CI, 1.38 to 4.03, P = 0.002), compared with those in the lowest risk group (high EPA and low transfat) (Figure 1).

**Table 5:** Combination of two fatty acids adjusted confounding factor in multivariable adjusted models

		Fatty Acid 1					Fatty Acid 2			
Model	Marker	Odds ratio	95% CI		P trend	Marker	Odds ratio	95% CI		P trend
1	EPA, % fat	0.55	0.35	0.86	0.039	Saturated fat, % fat	1.56	1.00	2.45	0.035
2	EPA, % fat	0.49	0.31	0.76	0.027	MUFA, % fat	2.07	1.29	3.32	0.001
3	EPA, % fat	0.52	0.33	0.81	0.057	Transfat, % fat	1.38	0.89	2.14	0.004
4	EPA, % fat	0.48	0.31	0.76	0.012	N-6 fatty acids, % fat	0.36	0.22	0.59	< .0001
5	EPA, % fat	0.63	0.38	1.06	0.43	DHA, % fat	0.72	0.42	1.21	0.033
6	DHA, % fat	0.65	0.39	1.09	0.036	Saturated fat, % fat	1.28	0.77	2.12	0.41
7	DHA, % fat	0.37	0.23	0.61	< .0001	MUFA, % fat	3.01	1.81	5.02	< .0001
8	DHA, % fat	0.72	0.43	1.19	0.10	Transfat, % fat	1.23	0.75	2.01	0.06
9	DHA, % fat	0.73	0.45	1.16	0.076	N-6 fatty acids, % fat	0.41	0.25	0.69	0.001
10	N-6 fatty acids, % fat	0.22	0.11	0.46	0.000	Saturated fat, % fat	0.60	0.30	1.17	0.17
11	N-6 fatty acids, % fat	0.24	0.14	0.41	< .0001	MUFA, % fat	3.02	1.82	5.02	< .0001
12	N-6 fatty acids, % fat	0.33	0.16	0.68	0.008	Transfat, % fat	0.78	0.41	1.50	0.88
13	Transfat, % fat	1.63	0.90	2.92	0.032	Saturated fat, % fat	1.00	0.54	1.84	0.99
14	Transfat, % fat	2.98	1.82	4.90	< .0001	MUFA, % fat	4.11	2.37	7.11	< .0001
15	MUFA, % fat	9.25	4.68	18.3	< .0001	Saturated fat, % fat	7.77	4.14	14.6	< .0001

Abbreviations as for Table 1

Results are from separate models of each combination of fatty acid profiles. Odds ratios and 95% confidence intervals in the highest compared with the lowest quintile of each fatty acid. P for trend across quintiles of each profile, and each fatty acid profile was added to the model in quintile with 4 dummy variables. Adjusted for age, gender, BMI, smoking, drinking and exercise, LDL cholesterol, systolic and diastolic blood pressure, uric acid, fasting glucose levels and total fat amount in plasma

## Discussion

Our study clearly demonstrated that plasma fatty acid components improved the prediction of the risk of the metabolic syndrome among ethnic Chinese adults in Taiwan. Significant simultaneous effects and joint additive effects were also shown for these fatty acids. Additional information on fatty acids is necessary to predict the status of metabolic syndrome in adult populations, implied by a good performance measure. Extrapolations to the clinical purpose of these findings were feasible for management of metabolic syndrome in primary prevention setting. Because of high prevalence of metabolic syndrome worldwide, our findings would be applied in clinical setting.

Our findings were consistent with previous studies. Both n-6 and n-3 polyunsaturated fatty acids have been associated with lower cardiovascular risk [11,12]. Dietary fat quality has been reported to influence the activity of enzymes involved in the desaturation of fatty acids in the body, and data from metabolic and clinical studies support recommendations to replace saturated and trans fat with unsaturated fat in the prevention of cardiovascular disease. Our data support that saturated fat, monounsaturated fat, and transfat are significantly associated with the risk of metabolic syndrome. For the protective role of fatty acids, marine fatty acids, especially EPA and DHA, have been shown to play a protective role in the risk of metabolic syndrome [12,13].

Our approach, integrating all fatty acid measurements instead of just one specific fatty acid, extended the understanding of the role of fatty acids on the risk of metabolic syndrome. After considering other fatty acids, DHA and EPA still played an important role for reducing the risk of metabolic syndrome status. The inverse relationship between marine n-3 fatty acids and metabolic syndrome may implicate the protective effect of n-3 PUFAs intake in isocaloric substitution for other fats on metabolic syndrome, which was associated with the increased risk of cardiovascular diseases[14]. Observational studies and clinical trials have shown that intake of EPA and DHA was associated to lower the cardiovascular risk[11]. The effects of n-3 fatty acids metabolic syndrome on cardiovascular disease were mediated through reducing some intermediate markers, such as metabolic syndrome components and inflammatory biomarkers [15]. The protective roles of EPA and DHA for the risk of metabolic syndrome might provide the early prevention of cardiovascular events. Our study clearly demonstrated the additional beneficial effects of EPA and DHA for the risk of the metabolic syndrome.

### Study strength and limitations

To the best of our knowledge, this study was one of the few studies to examine the cumulative effects of fatty acids on the risk of metabolic syndrome. The study sample size was relatively large and the characteristics of the study participants were homogeneous; so the results were internally consistent. However, several limitations should be mentioned. First, the fatty acids were only measured once, and variations due to the seasonal availability of foods were not evaluated. However, the non-differential misclassification due to measurement would underestimate the true effect. We believe the true association between fatty acids and the risk of metabolic syndrome may be more significant. Second, we did not collect the dietary habits in this study. However, biochemical measurements of fatty acids have a reasonable validity with the information from food frequency questionnaires [16,17].

## Conclusion

In conclusion, plasma fatty acid concentrations improved the prediction of the risk of metabolic syndrome. Additional joint effects were also found between two fatty acid levels. Among them, n-6 fatty acid and marine derived n-3 fatty acids, i.e., EPA and DHA, were protective factors for the risk of metabolic syndrome. Further studies on the mechanism of various fatty acids on the pathway of metabolic syndrome are warranted.

## Methods

### Study design and population

The study design was a nested case-control study design based on 8,911 adult participants who were recruited from the Health Management Center of one tertiary hospital from January 2004 to December 2007. All participants provided written informed consent with the study protocol being reported elsewhere [18-20]. In brief, details of socioeconomic status, along with medical and medication histories were collected by questionnaires, and standardized clinical procedures were undertaken. We excluded the participants with concurrent severe medical diseases such as cancer and heart failure. The participants signed informed consent forms, and the protocol was approved by the Institutional Research Board of the hospital.

Details of the subjects' medical histories such as medication, hospitalization and smoking status were included in the structural questionnaires. Standardized procedures for the physical examination, such as anthropometric measures and blood pressure, were performed [18,19]. Blood pressure was measured in a resting position by trained medical assistants. We defined the lifestyle and clinical covariates as smoking (current smoker, ever-smoker and never smoker), alcohol intake (regular/no), occupation (business and service work), regular exercise (yes/no), family history of coronary heart disease (yes/no), baseline hypertension (yes/no, defined by a blood pressure of at least 140/90 mm Hg or on medication), and diabetes mellitus (yes/no, defined by a fasting plasma glucose of at least 126 mg/dl or on medication).

### Blood sampling and analytic methods

The procedures for blood sampling and analytic methods have been described in previous studies [18,21]. In brief, blood samples were collected from each participant after fasting for at least 12 hours. Serum total cholesterol levels were measured using the CHOD-PAP method (Boehringer Mannheim, Germany). HDL-C was measured following precipitation of apolipoprotein B-containing lipoproteins with phosphotungstic acid and magnesium ions (Boehringer Mannheim, Germany). Triglyceride concentrations were measured by the GPO-DAOS method (Wako Co., Japan). All of the lipids were measured using a Hitachi 7450 automated analyzer (Hitachi, Japan). LDL-C concentrations were calculated using the Friedewald formula. Plasma fasting glucose and 2-hr postprandial glucose concentrations were measured using a Hitachi 7450 automated analyzer (Hitachi, Japan). HbA1c levels were measured using a DCA 2000 analyzer (Bayer Diagnostics, Elkhart, IN). All of the measurements were carried out in a single hospital. The coefficient of variation was 5%.

### Case ascertainment and matched control selection

Metabolic syndrome was defined according to the updated criteria of the Adult Treatment Panel III [22] and International Diabetes Federation (IDF), which have been shown to have excellent consistency [18]. We used the IDF criteria to ascertain the metabolic syndrome cases as those who had a major component of central obesity (defined by a waist circumference greater than 90 cm in men and 80 cm in women) and two other components from the following: 1) blood pressure of at least 130/85 mmHg or undergoing treatment for hypertension; 2) serum triglycerides of at least 1.7 mmol/L (150 mg/dL); 3) HDL cholesterol less than 1.0 mmol/L (40 mg/dL) in men and less than 1.3 mmol/L (50 mg/dL) in women; and 4) fasting glucose > = 5.5 mmol/L (100 mg/dL). In all, 1869 individuals (21%) were classified as having metabolic syndrome. Next, we randomly selected 1000 of these individuals and then control subjects were frequency-matched according to age and gender.

### Fatty acid profile measurements in gas chromatography

A 10-ml tube of EDTA-anticoagulated blood was collected, refrigerated at the site centers, and sent back within 3 hours to the National Taiwan University Hospital core laboratory. The blood was centrifuged at 800 × *g *for 10 min, then plasma was separated and dispensed into several aliquots and frozen at -70°C for analysis for fatty acid content by a single technician. After thawing, 0.5 mL of plasma was extracted with 0.5 mL methanol followed by 1.0 mL chloroform under a nitrogen atmosphere, and the lipid extract was filtered to remove protein. The methyl esters were then separated and measured on a 5890 gas chromatograph (Hewlett Packard, Avondale, PA) equipped with a 30m-FFAT WCOT glass capillary column (J & W Scientific, Folsom, CA) and a flame-ionization detector. The identities of 29 individual fatty acid peaks were ascertained by comparing each peak's retention time relative to the retention times of FAs of synthetic standards of known FA components. The relative amount of each FA (% of total FAs) was quantified by integrating the area under the peak, and dividing the result by the total area for all FAs. To minimize transcription errors, the data from the gas chromatogram was electronically transferred to a computer for data analysis.

### Statistical analysis

All data were presented as mean and standard deviation for continuous variables and contingency tables for categorical data, and were listed by status of case patients and control subjects. The age and gender adjusted Spearman correlation coefficients were examined. To compare different fatty acids in predicting the risk of metabolic syndrome, we used the following strategies:

First, the frequency of each fatty acid in cases and controls was calculated and stratified by fatty acid quintiles. We analyzed the association between various fatty acids and the risk of metabolic syndrome using the logistic regression model, adjusting for potential confounding factors. We specified three logistic models to evaluate the adjusted odds ratios of quintile values. Model 1 was adjusted for age (continuous variable) and gender only. Model 2 included additional body mass index (continuous variable), smoking, alcohol intake and exercise status. Model 3 included additional continuous LDL cholesterol, systolic and diastolic blood pressure, uric acid, fasting glucose levels and total fat amount in plasma. To test for linear trends across fatty acid categories, we used the median fatty acid profile levels within quintiles as a continuous variable. We also tested the goodness of fit of the model using the Hosmer and Lemeshow test [23], and the model fit was acceptable.

Second, we compared the performance of the models with and without fatty acid measurements using the area under the receiver operating characteristic curve (AUC). The curve is a graph of sensitivity versus 1-specificity (or false-positive rate) for various cutoff definitions of a positive diagnostic test result [24]. Statistical differences in the AUCs were compared using the method of DeLong *et al. *[25], and the difference of AUC was used to compare the discriminatory capability among the models.

Third, we provided several additional statistics, including integrated discrimination improvement (IDI) and net reclassification improvement (NRI) [26] for the comparison of nested models with and without fatty acids, because the AUC value is not the best discriminatory statistic for prediction power [26-28]. In brief, NRI was based on the reclassification tables and was calculated from a sum of differences between the 'upward' movement in categories for event subjects and the "downward' movement in those for nonevent subjects [26]. A priori risk categories were defined according to the a priori risk categories of stroke (0-10%, 10-50%, 50-90%, and > = 90%), and the reclassification tables according to the metabolic syndrome were specified. The IDI is considered to be the difference between improvement in average sensitivity and any potential increase in average 'one minus specificity' [26], and is estimated as the difference in Yates discrimination slopes between the nested models [29,30]. The reclassification table as a tool for comparing models was suggested by Ridker and colleagues [28].

All statistical analyses were performed using SAS version 9.1 (SAS Institute, Inc., Cary, NC) and STATA version 9.1 (Stata Corp., College Station, Texas).

## Competing interests

The authors declare that they have no competing interests.

## Authors' contributions

KLC, YTL and MFC proposed the study design. KLC, CLC, CHK, HJL, and BCL participated in data collection. KLC and PHL performed statistical and genetic analysis. PRC provided consultation and modified the draft. HCH performed fatty acid measurements and quality control. YTL and MFC conceived of the study, and participated in its design and coordination and helped to draft the manuscript. All authors read and approved the final manuscript.

## Supplementary Material

Additional file 1 **Additional Tables S1-S3**. Including supplementary tables for Spearman partial correlation coefficients, Adjusted odds ratios and 95% confidence intervals for fatty acid concentrations, specified by continuous variables, and Net reclassification tables. Click here for file
