# Supplementary material for: Plasma fatty acids and the risk of metabolic syndrome in ethnic Chinese adults in Taiwan
Source: Lipids Health Dis. 2011 Feb 21;10:33. doi: 10.1186/1476-511X-10-33 (PMC3056817; doi:10.1186/1476-511X-10-33)
Supplement: Additional file 1 — Additional Tables S1-S3. Including supplementary tables for Spearman partial correlation coefficients, Adjusted odds ratios and 95% confidence intervals for fatty acid concentrations, specified by continuous variables, and Net reclassification tables. [file 1476-511X-10-33-S1.DOC]

**Additional file 1, Table S1: Age- and gender-adjusted Spearman partial correlation coefficients between clinical measures and fatty acid profiles among the study participants, bold numbers indicating *P*<0.05**

|  | Systolic BP | Diastolic BP | BMI | Waist | Total cholesterol | Triglycerides | HDL cholesterol | LDL cholesterol | Uric acid | Fasting glucose | Glomerular filtration rate |
| --- | --- | --- | --- | --- | --- | --- | --- | --- | --- | --- | --- |
| Fatty acid, mg/dL |  |  |  |  |  |  |  |  |  |  |  |
| Saturated fat | 0.10 | **0.10** | **0.21** | **0.23** | **0.44** | **0.66** | 0.00 | **0.29** | **0.13** | **0.19** | -0.04 |
| MUFA | **0.14** | **0.15** | **0.22** | **0.24** | **0.38** | **0.74** | **-0.10** | **0.18** | **0.17** | **0.14** | -0.04 |
| Trans fatty acid | **0.11** | **0.10** | **0.21** | **0.22** | **0.29** | **0.62** | -0.08 | **0.18** | **0.13** | **0.16** | -0.03 |
| PUFA | 0.06 | **0.09** | 0.07 | 0.07 | **0.52** | **0.51** | 0.08 | **0.30** | **0.10** | 0.08 | -0.04 |
| N-6 fatty acids | 0.05 | 0.09 | 0.08 | 0.08 | **0.50** | **0.51** | 0.06 | **0.30** | **0.09** | 0.08 | -0.04 |
| N-3 fatty acids | 0.07 | **0.11** | 0.04 | 0.03 | **0.44** | **0.32** | **0.16** | **0.21** | **0.09** | 0.06 | 0.00 |
| Marine fatty acids | 0.06 | **0.09** | 0.01 | 0.00 | **0.36** | **0.21** | **0.19** | **0.16** | 0.07 | 0.05 | 0.01 |
| EPA | 0.01 | 0.05 | -0.01 | -0.01 | **0.21** | 0.02 | **0.21** | **0.12** | 0.04 | 0.07 | 0.00 |
| DHA | 0.06 | **0.09** | 0.01 | 0.00 | **0.37** | **0.24** | **0.16** | **0.16** | 0.07 | 0.04 | 0.01 |
| Fatty acid, % total fatty acids | | | |  |  |  |  |  |  |  |  |
| Total fat amount | **0.11** | **0.12** | **0.20** | **0.21** | **0.51** | **0.73** | 0.00 | **0.31** | **0.15** | **0.17** | -0.05 |
| Saturated fat | 0.02 | 0.00 | **0.10** | **0.12** | 0.05 | **0.15** | 0.01 | 0.08 | 0.01 | **0.14** | -0.01 |
| MUFA | **0.12** | **0.12** | **0.16** | **0.18** | 0.05 | **0.44** | **-0.20** | -0.08 | **0.13** | 0.03 | 0.00 |
| Trans fatty acid | 0.05 | 0.02 | **0.11** | **0.12** | -0.04 | **0.22** | **-0.12** | -0.02 | 0.04 | 0.07 | 0.00 |
| PUFA | **-0.10** | -0.07 | **-0.22** | **-0.24** | -0.05 | **-0.44** | **0.13** | -0.02 | **-0.09** | **-0.17** | 0.02 |
| N-6 fatty acids | **-0.11** | -0.08 | **-0.21** | **-0.23** | -0.06 | **-0.42** | **0.09** | -0.01 | **-0.10** | **-0.17** | 0.01 |
| N-3 fatty acids | -0.02 | 0.00 | **-0.13** | **-0.16** | 0.03 | **-0.29** | **0.16** | -0.01 | -0.03 | -0.08 | 0.04 |
| Marine fatty acids | -0.03 | 0.00 | **-0.12** | **-0.14** | 0.02 | **-0.27** | **0.18** | -0.02 | -0.03 | -0.06 | 0.04 |
| EPA | -0.04 | -0.01 | **-0.09** | **-0.10** | 0.00 | **-0.28** | **0.21** | 0.01 | -0.03 | -0.01 | 0.03 |
| DHA | -0.02 | 0.00 | **-0.12** | **-0.14** | 0.03 | **-0.24** | **0.16** | -0.02 | -0.03 | -0.07 | 0.04 |

Abbreviations see Table 1.

**Additional file 1, Table S**2: Adjusted odds ratios and 95% confidence intervals for fatty acid concentrations, specified by continuous variables

|  | Unit as concentration | |  |  |  |  |  | Unit as standard deviation | | |  |
| --- | --- | --- | --- | --- | --- | --- | --- | --- | --- | --- | --- |
|  | Odds ratio | 95% CI | | Mean | SD | Estimated coefficient | SE | Odds ratio | 95% CI | | P |
| Saturated fat, % fat | 1.03 | 1.00 | 1.07 | 42.4 | 4.6 | 0.15 | 0.07 | 1.16 | 1.01 | 1.33 | 0.042 |
| MUFA, % fat | 1.08 | 1.02 | 1.14 | 15.3 | 2.7 | 0.20 | 0.08 | 1.23 | 1.05 | 1.43 | 0.010 |
| Transfat, % fat | 1.09 | 1.01 | 1.18 | 6.74 | 1.93 | 0.16 | 0.08 | 1.18 | 1.01 | 1.37 | 0.033 |
| N-6 fatty acids, % fat | 0.94 | 0.90 | 0.97 | 31.1 | 4.4 | -0.29 | 0.08 | 0.75 | 0.64 | 0.87 | 0.0001 |
| EPA, % fat | 0.53 | 0.33 | 0.84 | 0.50 | 0.33 | -0.21 | 0.08 | 0.81 | 0.70 | 0.95 | 0.0075 |
| DHA, % fat | 0.81 | 0.69 | 0.94 | 2.64 | 0.96 | -0.20 | 0.07 | 0.82 | 0.70 | 0.94 | 0.006 |

Adjusted for age, gender, BMI, smoking, drinking and exercise, LDL cholesterol, systolic and diastolic blood pressure, uric acid, fasting glucose levels and total fat amount in plasma; SD, standard deviation; SE, standard error.

**Additional file 1, Table S3: Net reclassification tables of the models with and without EPA and transfat concentrations in the model:**

**For EPA level**

|  | Additional model | |  |  |  |
| --- | --- | --- | --- | --- | --- |
| Controls | Lowest | Low | Medium | High | Total |
| Baseline |  |  |  |  |  |
| Lowest | 445 | 16 | 0 | 0 | 461 |
| Low | 13 | 360 | 7 | 0 | 380 |
| Medium | 0 | 11 | 120 | 0 | 131 |
| High | 0 | 0 | 5 | 14 | 19 |
| Total | 458 | 387 | 132 | 14 | 991 |
| Cases | Additional model | |  |  |  |
| Baseline | Lowest | Low | Medium | High | Total |
| Lowest | 17 | 1 | 0 | 0 | 18 |
| Low | 1 | 134 | 8 | 0 | 143 |
| Medium | 0 | 10 | 333 | 18 | 361 |
| High | 0 | 0 | 5 | 473 | 478 |
| Total | 18 | 145 | 346 | 491 | 1000 |

**For transfat level**

|  | Additional model | |  |  |  |
| --- | --- | --- | --- | --- | --- |
| Controls | Lowest | Low | Medium | High | Total |
| Baseline |  |  |  |  |  |
| Lowest | 447 | 14 | 0 | 0 | 461 |
| Low | 17 | 350 | 13 | 0 | 380 |
| Medium | 0 | 10 | 120 | 1 | 131 |
| High | 0 | 0 | 5 | 14 | 19 |
| Total | 464 | 374 | 138 | 15 | 991 |
| Cases | Additional model | |  |  |  |
| Baseline | Lowest | Low | Medium | High | Total |
| Lowest | 14 | 4 | 0 | 0 | 18 |
| Low | 1 | 130 | 12 | 0 | 143 |
| Medium | 0 | 8 | 332 | 21 | 361 |
| High | 0 | 0 | 11 | 467 | 478 |
| Total | 15 | 142 | 355 | 488 | 1000 |
